# Associations between reporting of cancer alarm symptoms and socioeconomic and demographic determinants: a population-based, cross-sectional study

**DOI:** 10.1186/1471-2458-12-686

**Published:** 2012-08-22

**Authors:** Rikke Pilsgaard Svendsen, Maja Skov Paulsen, Pia Veldt Larsen, Bjarne Lühr Hansen, Henrik Støvring, Dorte Ejg Jarbøl, Jens Søndergaard

**Affiliations:** 1 Research Unit of General Practice, Institute of Public Health, University of Southern Denmark, J. B. Winsløwsvej 9A, DK-5000, Odense C, Denmark; 2 Department of Public Health, Biostatistics, University of Aarhus, Bartholins Allé 2, Bldg 1261, DK-8000, Aarhus, Denmark

**Keywords:** Breast cancer, Colorectal cancer, Cross-sectional survey, Lung cancer, Socioeconomic factors, Signs and symptoms, Urinary tract cancer

## Abstract

**Background:**

Reporting of symptoms which may signal cancer is the first step in the diagnostic pathway of cancer diseases. Cancer alarm symptoms are common in the general population. Public awareness and knowledge of cancer symptoms are sparse, however, and many people do not seek medical help when having possible cancer symptoms. As social inequality is associated with cancer knowledge, cancer awareness, and information-seeking, our hypothesis is that social inequality may also exist in the general population with respect to reporting of cancer alarm symptoms. The aim of this study was to investigate possible associations between socioeconomic and demographic determinants and reporting of common cancer alarm symptoms.

**Methods:**

A cross-sectional questionnaire survey was performed based on a stratified sample of the Danish general population. A total of 13 777 randomly selected persons aged 20 years and older participated. Our main outcome measures were weighted prevalence estimates of self-reporting one of the following cancer alarm symptoms during the preceding 12 months: a lump in the breast, coughing for more than 6 weeks, seen blood in urine, or seen blood in stool. Logistic regression models were used to calculate unadjusted and adjusted odds ratios with 95% confidence intervals for the associations between each covariate and reporting of cancer alarm symptoms.

**Results:**

A total of 2 098 (15.7%) of the participants reported one or more cancer alarm symptoms within the preceding 12 months.

Women, subjects out of the workforce, and subjects with a cancer diagnosis had statistically significantly higher odds of reporting one or more cancer alarm symptoms. Subjects with older age and subjects living with a partner had lower odds of reporting one or more cancer alarm symptoms. When analysing the four alarm symptoms of cancer separately most tendencies persisted.

**Conclusions:**

Socioeconomic and demographic determinants are associated with self-reporting of common cancer alarm symptoms.

## Background

Reporting of symptoms which may signal cancer is the first step in the diagnostic pathway of cancer diseases [1]. Some cancer symptoms are quite unspecific while others are more characteristic and distinctive – so-called cancer ‘alarm symptoms’.

Cancer alarm symptoms are common in the general population [2]. In a Danish population-based study a total of 15% reported having experienced at least one of four common cancer alarm symptoms [3], and 18% of the Australian population reported blood in the stools during a 12-month period [4]. However, public awareness and knowledge of cancer symptoms are sparse and many people do not seek medical help when experiencing cancer symptoms. Hence, increasing focus is on raising awareness in the population of early symptoms of cancer in order to increase the ability to notice and report alarm symptoms [5-8].

Socioeconomic inequalities in health are ubiquitous and relations between socioeconomic status and morbidity and mortality seem to persist for numerous diseases, including many cancers [9]. Further, social disparities are significantly associated with different information-seeking behaviours among cancer patients [10].

In Denmark, the majority of health services are free of charge. Still, socioeconomic differences persist for cancer incidence, time from experiencing a symptom until seeking medical help, and cancer survival [11]. As social inequality is associated with cancer knowledge, cancer awareness, and information-seeking, our hypothesis is that social inequality also exists with respect to reporting of cancer alarm symptoms.

The aim of this study was, in a population-based cross-sectional design, to investigate possible associations between socioeconomic and demographic determinants and self-reporting of frequent cancer alarm symptoms.

## Methods

### Study design

A cross-sectional questionnaire survey based on a stratified sample of the general population was conducted in April 2007 in the former County of Funen, Denmark, with approx. 480 000 inhabitants, comprising 9% of the total Danish population [12]. All Danish citizens are registered with the Danish Civil Registration System with a unique personal identification number, used in all national registers and enabling accurate linkage between all of them [13].

### Sampling

The survey comprised a questionnaire sent out to a sample of 20 000 people aged 20 years or older. The sample was randomly selected from the Danish Civil Registration System, stratified on gender and age, half of them women and half of them men, so that for each gender, only 1000 subjects under the age of 40 years were included. Further details of the survey are described elsewhere [3].

### Data sources and measurements

#### The questionnaire

The questionnaire concerned four types of cancer: breast, lung, urinary tract, and colorectal cancer. These four cancers were chosen because they are the most common cancer forms in Denmark [14] and because their symptoms are well described in the literature [15-18]. For each cancer type there was a question on whether the person had a specific symptom highly related to that particular cancer. Subjects were asked whether they within the preceding 12 months had: “Felt a lump in your breast?”, “Coughed for more than 6 weeks?”, “Seen blood in your urine?”, or “Seen blood in your stool?” They were further asked: “Do you have, or have you had, a cancer disease”? Answers to each question could be checked as a “yes” or a “no”. All symptoms reported in this paper are thus self-reported.

Before the questionnaire was sent out, it was tested: First 10 subjects were interviewed on its comprehensibility. Then the questionnaire was filled in twice by 200 subjects aged 40 years and older, with the objective of analysing how the questionnaire was perceived by recipients and to assess its reproducibility. The assessment led to minor changes.

### Outcome variables

A “yes” response to one of the listed symptoms was considered a positive response. The answer “no” and not answering an item were considered negative responses.

### Statistics Denmark and socioeconomic and demographic variables

All socioeconomic and demographic factors were collected by data linkage to Statistics Denmark using a person unique civil registration number. Statistics Demark is a governmental institution collecting information electronically provided by administrative registers of different governmental agencies [19]. We obtained information for each subject about a number of socioeconomic variables: educational level, income level and labour market affiliation. Furthermore, we obtained information on cohabitation status, as we believed this demographic factor to be important, when reporting cancer alarm symptoms. Information was retrieved for the year preceding the questionnaire (2006). To account for annual variation in income we calculated the average income for the preceding 5 years.

In order to compare our sample with the Danish general population and for calculating weighted estimates we retrieved data on sex, age, education, income and employment for the entire Danish population aged 20 years and older for the year 2006.

Education was categorised according to the highest attained educational level: < 10 years (primary and lower secondary school), 10–12 years (vocational education and upper secondary school), >12 years (short, medium and long-term higher education) [20-23]. We obtained gross income, comprising all income liable to general taxation (wages and salaries, all types of benefits and pensions) for each person. Income was categorised according to the 5-year average income as low income (1^st^ quartile), middle income (2^nd^ and 3^rd^ quartile), and high income (4^th^ quartile) [24]. Labour market affiliation was categorised into three groups: working; pensioners (early retirement pension and old-age pension); out of the workforce (receiving disability pension, social security, and being unemployed). Cohabitation status was categorised as living with a partner (married/cohabitating) or single (divorced, widowed or never married) [23].

### Statistical analysis

Prevalence estimates of reporting one or more alarm symptoms of cancer and prevalence estimates of reporting each specific alarm symptom of cancer in the population within the preceding 12 months were calculated. Estimates were reported as percentages (%) with 95% exact confidence intervals (CIs), based on binominal distributions.

“Logistic regression models were used to calculate unadjusted and adjusted odds ratios (ORs) with 95% CIs for the association between each covariate and reporting of cancer alarm symptoms. The covariates considered were: sex, age, education, income, affiliation to the labour market, cohabitation status [24], and having a cancer diagnosis. In the adjusted analyses adjustments were made for the a priori selected possible confounders: sex, age and having a cancer diagnosis [25,26]”.

All estimates for symptom prevalences were weighted according to the total Danish population to account for the stratified sampling procedure.

### Ethical considerations

According to the Act on a Biomedical Research Ethics Committee System the project was not a biomedical research project and therefore did not need the ethics committee’s approval, journal number 2011-41-6709. The study was approved by the Danish Data Protection Agency.

## Results

### Description of participants

**Figure 1:**
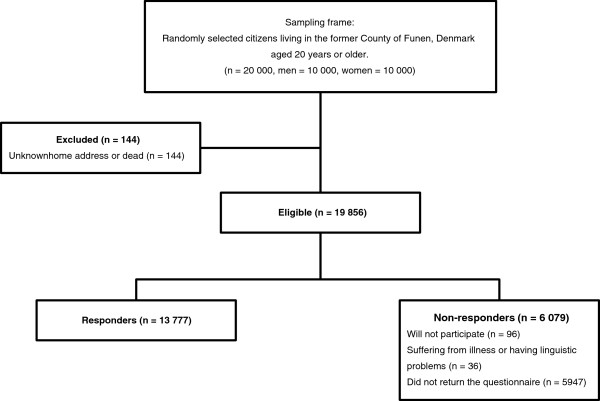
Study sample.

Of the 20 000 subject identified, 144 subjects (0.7%) were not eligible because they were either dead or could not be reached (Figure 1). Of the 19 856 eligible, 36 (0.2%) subjects could not participate because they were suffering from dementia or had language problems. Overall 13 777 subjects returned the questionnaire yielding a response rate of 69.4% (Figure 1). Table 1 shows the descriptive data of the participants (weighted and unweighted) and of the entire Danish population aged 20+.

**Table 1:** Descriptive data on study population and the Danish population

		**Total study population**		**Weighted total study population**	**Danish population**
		**n = 13 777**		**n = 13 777**	**n = 4 110 111**
		**n**	**% (95% CI)**	**% (95% CI)**	**%**
**Sex**	Men	6 533	47.4 (46.6 to 48.3)	45.8 (45.0 to 46.7)	48.9
	Women	7 244	52.6 (51.7 to 53.4)	54.2 (53.3 to 55.0)	51.1
**Age, years**	20-39	1 105	8.0 (7.6 to 8.5)	29.0 (28.2 to 29.8)	33.8
	40-59	6 403	46.5 (45.6 to 47.3)	35.9 (35.1 to 36.7)	37.1
	60-79	5 357	38.9 (38.1 to 39.7)	30.0 (29.2 to 30.8)	23.7
	80-99	912	6.6 (6.2 to 7.0)	5.1 (4.8 to 5.5)	5.5
**Educational level**	Low	4 136	31.0 (30.2 to 31.8)	26.6 (25.8 to 27.3)	31.2
	Medium	5 588	41.8 (41.0 to 42.7)	43.4 (42.6 to 44.3)	43.9
	High	3 631	27.2 (26.4 to 27.9)	28.9 (28.1 to 29.7)	24.9
**Income level**	Low	3 444	25 (24.3 to 25.7)	26.6 (25.8 to 27.3)	31.1
	Medium	6 888	50 (49.2 to 50.8)	50.7 (49.8 to 51.5)	49.1.
	High	3 444	25 (24.3 to 25.7)	22.7 (22.0 to 23.5)	19.8
**Labour market affiliation**	Working	7 989	59.1 (58.3 to 60.0)	66.4 (65.6 to 67.2)	64.3
	Pensioners	4 414	32.7 (31.9 to 33.5)	25.3 (24.6 to 26.1)	22.2
	Out of workforce	1 105	8.2 (7.7 to 8.6)	8.3 (7.8 to 8.7)	13.5
**Cohabitating status**	Single	3 760	27.3 (26.6 to 28.0)	29.4 (28.6 to 30.1)	-
	Cohabitant / married	10 013	72.7 (72.0 to 73.4)	70.6 (69.9 to 71.4)	-
**Cancer diagnosis**	No	12 531	91.0 (90.5 to 91.4)	92.6 (92.1 to 93.0)	-
	Yes	1 246	9.0 (8.6 to 9.5)	7.4 (7.0 to 7.9)	-

### Socioeconomic and demographic participant characteristics and reporting of one or more cancer alarm symptoms

Weighted prevalence estimates of reporting of one or more cancer alarm symptoms within the preceding 12 months together with crude and adjusted odds ratios for associations between symptom reporting and socioeconomic and demographic characteristics are presented in Table 2.

**Table 2:** Weighted prevalence estimates and Crude and adjusted odds ratios of reporting one or more cancer symptoms

		**Participants reporting of one or more symptoms**					
		**n = 2 098**	**weighted prevalence% (95% CI)**	**OR (crude)**	**95% CI**	OR (adjusted)*	**95% CI**
**Sex**	Men	908	40.7 (38.6 to 47.7)	1		1	
	Women	1 190	59.3 (57.2 to 61.4)	**1.22**	1.11 to 1.34	**1.19**	1.09 to 1.31
**Age, years**	20-39	191	31.9 (29.8 to 33.9)	1		1	
	40-59	1 090	39.0 (36.9 to 41.1)	0.98	0.83 to 1.16	0.96	0.81 to 1.14
	60-79	726	25.9 (24.0 to 27.8)	**0.75**	0.63 to 0.89	**0.71**	0.60 to 0.85
	80-99	91	3.3 (2.5 to 4.0)	**0.53**	0.41 to 0.69	**0.48**	0.37 to 0.63
**Educational level**	Low	634	27.7 (26.9 to 28.6)	1		1	
	Medium	852	42.7 (40.6 to 44.9)	0.99	0.88 to 1.11	0.94	0.84 to 1.06
	High	568	30.0 (28.0 to 32.0)	1.02	0.91 to 1.16	0.95	0.83 to 1.08
**Income level**	Low	496	26.7 (24.8 to 28.6)	1		1	
	Medium	1 081	51.7 (49.6 to 53.9)	1.11	0.99 to 1.24	0.98	0.87 to 1.11
	High	521	21.6 (19.8 to 23.3)	1.06	0.93 to 1.21	0.95	0.82 to 1.10
**Labour market affiliation**	Working	1 249	68.5 (66.4 to 70.5)	1		1	
	Pensioners	544	19.9 (18.2 to 21.7)	**0.76**	0.68 to 0.85	0.93	0.78 to 1.10
	Out of workforce	254	11.6 (10.2 to 13.0)	**1.61**	1.38 to 1.88	**1.59**	1.36 to 1.86
**Cohabitating status**	Single	618	30.4 (28.4 to 32.4)	1		1	
	Cohabitant / married	1 480	69.6 (67.6 to 71.6)	**0.88**	0.80 to 0.98	**0.84**	0.76 to 0.93
**Cancer diagnosis**	No	1 841	90.2 (88.9 to 91.5)	1		1	
	Yes	257	9.8 (8.5 to 11.1)	**1.51**	1.30 to 1.75	**1.63**	1.40 to 1.89

(*adjusted for sex, age and having a cancer diagnosis.) Highlighted figures are statistically significant (p <0.05).

A total of 2 098 participants (15.7%) reported one or more cancer alarm symptoms within the preceding 12 months. The mean age of respondents who reported one or more cancer alarm symptoms was 49.7 years and 59.3% were women (all weighted estimates).

The adjusted analyses showed that women had statistically significantly higher odds of reporting a symptom of cancer, as did subjects out of the workforce, and subjects with a cancer diagnosis. Those aged 60–79, those aged 80–99, and those living with a partner, had statistically significantly lower odds of reporting alarm symptoms. Education and income were not statistically significantly associated with reporting of one or more cancer alarm symptoms.

### Socioeconomic and demographic participant characteristics and prevalence of reporting each specific cancer alarm symptom

Weighted prevalence estimates of reporting each specific alarm symptom of cancer within the preceding 12 months and socioeconomic and demographic characteristics are presented in Table 3.

**Table 3:** Weighted prevalence estimates of participant characteristics by reporting a specific cancer symptom within the preceding 12 months

		**Weighted prevalences**							
		**Felt a lump in the breast yes, n = 411**		**Coughed for more than six weeks yes, n = 940**		**Seen blood in urine yes, n = 307**		**Seen blood on stools yes, n = 713**	
		**n**	**% (95% CI)**	**n**	**% (95% CI)**	**n**	**% (95% CI)**	**n**	**% (95% CI)**
**Sex**	Men	66	13.4 (10.2 to 16.6)	412	43.1 (39.8 to 46.4)	149	42.7 (40.0 to 48.5)	402	54.4 (50.9 to 58.0)
	Women	345	86.6 (83.4 to 89.8)	528	56.8 (53.6 to 60.2)	158	57.3 (51.5 to 63.0)	311	45.6 (42.0 to 49.1)
**Age, years**	20-39	46	36.5 (32.0 to 41.1)	60	23.7 (20.9 to26.6)	21	25.1 (20.0 to 30.1)	88	39.1 (35.7 to 42.6)
	40-59	259	45.1 (40.4 to 49.8)	441	38.2 (35.0 to 41.5)	120	31.4 (26.0 to 36.8)	407	39.6 (36.2 to 43.1)
	60-79	89	15.4 (12.0 to 18.8)	399	34.6 (31.4 to 37.8)	142	37.2 (31.5 to 42.8)	196	19.1 (16.3 to 21.9)
	80-99	17	2.9 (1.3 to 4.5)	40	3.5 (2.2 to 4.7)	24	6.3 (3.4 to 9.1)	22	2.1 (1.1 to 3.2)
**Educational level**	Low	120	25.5 (21.4 to 29.7)	329	34.2 (31.0 to 37.4)	93	27.3 (22.0 to 32.6)	186	22.2 (19.2 to 25.2)
	Medium	152	40.5 (35.8 to 45.2)	381	42.7 (39.3 to 46.0)	133	49.9 (43.8 to 55.8)	290	42.6 (39.1 to 46.2)
	High	132	34.0 (29.5 to 38.5)	202	23.2 (20.3 to 26.0)	70	22.9 (17.9 to 27.9)	225	35.2 (31.8 to 38.6)
**Income level**	Low	86	26.1 (21.9 to 30.2)	261	28.2 (25.2 to 31.2)	87	32.1 (26.7 to 37.6)	148	26.2 (23.1 to 29.4)
	Medium	226	53.6 (48.9. to 58.3)	480	52.4 (49.1 to 55.8)	152	47.3 (41.4 to 53.1)	357	49.7 (46.1 to 53.3)
	High	99	20.3 (16.5 to 24.1)	199	19.4 (16.8 to 22.1)	68	20.6 (15.9 to 25.3)	208	24.1 (21.0 to 27.1)
**Labour market affiliation**	Working	283	76.0 (72.0 to 80.1)	481	60.0 (56.7 to 63.3)	157	60.6 (54.8 to 66.3)	472	74.6 (71.4 to 77.7)
	Pensioners	69	12.2 (9.0 to15.3)	289	25.7 (22.8 to 28.7)	119	31.6 (26.1 to 37.0)	137	13.7 (11.3 to 16.2)
	Out of workforce	49	11.8 (8.7 to 14.8)	142	14.2 (11.9 to 16.6)	26	7.8 (4.7 to 11.0)	88	11.7 (9.4 to 14.0)
**Cohabitation status**	Single	121	30.3 (26.0 to 34.6)	302	32.7 (29.5 to 35.8)	102	37.9 (32.2 to 43.6)	190	25.8 (22.7 to 28.9)
	Cohabitant / married	290	69.7 (65.4 to 74.0)	638	67.3 (64.2 to 70.4)	205	62.1 (56.4 to 67.8)	523	74.2 (71.1 to 77.3)
**Cancer diagnosis**	No	331	85.5 (82.1 to 88.8)	842	91.5 (89.6 to 93.4)	257	85.0 (80.9 to 89.2)	632	91.1 (89.0 to 93.1)
	Yes	80	14.5 (11.2 to 17.9)	98	8.5 (6.6 to 10.4)	50	15.0 (10.8 to 19.1)	81	8.9 (6.9 to 11.0)

A total of 411 subjects (3.3%) had felt a lump in the breast; 940 subjects (6.5%) had coughed for more than 6 weeks; 307 subjects (2.1%) reported having seen blood in the urine, and 713 subjects (5.8%) reported having seen blood in the stool within the preceding 12 months (weighted prevalences).

Crude and adjusted odds ratios of associations between reporting a specific alarm symptom within the preceding 12 months and socioeconomic and demographic participant characteristics are presented in Table 4. Only results from the adjusted analyses are presented in the manuscript.

**Table 4:** Unadjusted and adjusted odds ratios of reporting a specific cancer symptom within the preceding 12 months by participant characteristics

		**Felt a lump in the breast**				**Coughed for more than six weeks**				**Seen blood in urine**				**Seen blood in stool**			
		**Crude**	**95% CI**	Adjusted*	**95% CI**	**Crude**	**95% CI**	Adjusted*	**95% CI**	**Crude**	**95% CI**	Adjusted* 95% CI		**Crude 95% CI**		Adjusted* 95% CI	
**Sex**	male	1		1		1		1		1		1		1		1	
	Female	**4.90**	3.76 to 6.39	**4.63**	3.54 to 6.04	**1.17**	1.02 to 1.33	**1.17**	1.02 to 1.34	0.96	0.76 to 1.20	0.93	0.74 to 1.17	**0.68**	0.59 to 0.80	**0.66**	0.57 to 0.77
**Age, years**	20-39	1		1		1		1		1		1		1		1	
	40-59	0.97	0.70 to 1.34	0.91	0.66 to 1.26	1.29	0.98 to 1.70	1.28	0.97 to 1.69	0.99	0.62 to 1.57	0.95	0.59 to 1.51	**0.78**	0.62 to 1.00	**0.76**	0.60 to 0.96
	60-79	**0.39**	0.27 to 0.56	**0.34**	0.23 to 0.49	**1.40**	1.06 to 1.85	**1.39**	1.05 to 1.84	1.41	0.88 to 2.23	1.29	0.81 to 2.05	**0.44**	0.34 to 0.57	**0.40**	0.31 to 0.53
	80-99	**0.44**	0.25 to 0.77	**0.33**	0.18 to 0.58	0.80	0.53 to 1.20	0.77	0.51 to 1.17	1.40	0.77 to 2.52	1.22	0.67 to 2.22	**0.29**	0.18 to 0.46	**0.26**	0.16 to 0.42
**Educational level**	Low	1		1		1		1		1		1		1		1	
	Medium	0.94	0.73 to 1.19	0.88	0.69 to 1.14	**0.85**	0.73 to 0.99	0.86	0.73 to 1.00	1.06	0.81 to 1.39	1.14	0.87 to 1.50	1.16	0.96 to 1.40	0.96	0.79 to 1.16
	High	1.26	0.98 to 1.62	1.06	0.82 to 1.38	**0.68**	0.57 to 0.82	**0.69**	0.57 to 0.83	0.85	0.62 to 1.17	0.94	0.68 to 1.30	**1.40**	1.15 to 1.71	1.14	0.93 to 1.40
**Income level**	Low	1		1		1		1		1		1		1		1	
	Medium	**1.32**	1.03 to 1.70	1.05	0.80 to 1.38	0.91	0.78 to 1.07	0.86	0.73 to 1.02	0.87	0.67 to 1.14	0.96	0.73 to 1.28	**1.22**	1.00 to 1.48	0.95	0.77 to 1.69
	High	1.16	0.86 to 1.55	1.28	0.92 to 1.78	**0.75**	0.62 to 0.91	**0.71**	0.57 to 0.88	0.78	0.56 to 1.07	0.89	0.62 to 1.28	**1.43**	1.15 to 1.79	0.93	0.73 to 1.19
**Labour market affiliation**	Working	1		1		1		1		1		1		1		1	
	Pensioners	**0.43**	0.33 to 0.56	0.66	0.42 to 1.03	1.09	0.94 to 1.27	1.06	0.85 to 1.33	**1.38**	1.08 to 1.76	1.05	0.72 to 1.52	**0.51**	0.42 to 0.62	0.80	0.60 to 1.08
	Out of workforce	1.26	0.93 to 1.72	1.10	0.80 to 1.52	**2.30**	1.89 to 2.81	**2.10**	1.72 to 2.57	1.02	0.79 to 1.83	1.30	0.88 to 1.93	**1.38**	1.09 to 1.75	**1.51**	1.18 to 1.91
**Cohabit status**	Single	1		1		1		1		1		1		1		1	
	Cohabitant / married	0.90	0.72 to 1.11	0.91	0.72 to 1.14	**0.78**	0.68 to 0.90	**0.74**	0.64 to 0.86	**0.75**	0.59 to 0.95	**0.77**	0.60 to 0.98	1.04	0.87 to 1.23	0.93	0.78 to 1.11
**Cancer diagnosis**	No	1		1		1		1		1		1		1		1	
	Yes	**2.53**	1.97 to 3.25	**2.73**	2.14 to 3.55	1.19	0.95 to 1.47	1.17	0.94 to 1.46	**2.00**	1.47 to 2.72	**1.90**	1.39 to 2.60	**1.31**	1.03 to 1.66	**1.68**	1.31 to 2.14

(*adjusted for sex, age and having a cancer diagnosis.) Highlighted figures are statistically significant (p <0.05).

### Gender

Women had statistically significantly higher odds than men of reporting a lump in the breast and of reporting coughing for more than 6 weeks, but had statistically significantly lower odds of reporting blood in the stool.

### Age

Subjects aged 60–79 years had statistically significantly higher odds of reporting coughing. Subjects with older age had statistically significantly lower odds of reporting a lump in the breast and of reporting blood in the stool within the preceding 12 months.

### Education

Subjects with high educational level had statistically significantly lower odds of reporting coughing for more than 6 weeks within the preceding 12 months than those with a low educational level.

### Income

Analyses showed a tendency towards subjects with increasing income having lower odds of reporting alarm symptoms (apart from having felt a lump in the breast) than those with low income. Results were only statistically significant for reporting coughing for more than 6 weeks within the preceding 12 months.

### Labour market affiliation

Those out of the workforce were statistically significantly associated with reporting coughing for more than 6 weeks and reporting blood in the stools within the preceding 12 months.

### Cohabitating status

For all four cancer alarm symptoms there was a tendency towards lower odds of symptom reporting for those living with a partner than for those being single. Results were statistically significant with respect to coughing and seeing blood in the urine.

### Having cancer

For all four cancer alarm symptoms there was a tendency towards higher odds of reporting cancer alarm symptoms for subjects with a cancer diagnosis. Results were not statistically significant of reporting coughing for more than 6 weeks.

## Discussion

In this large population-based survey socioeconomic and demographic factors were associated with reporting of common cancer alarm symptoms. Some 15.7% of the participants reported having experienced one or more cancer alarm symptoms within the preceding 12 months.

Women, subjects out of the workforce, and subjects with a cancer diagnosis had statistically significantly higher odds of reporting one or more cancer alarm symptoms. Subjects with older age and subjects living with a partner had statistically significantly lower odds of reporting one or more cancer alarm symptoms. When analysing each cancer alarm symptom separately, most tendencies persisted.

### Strengths and limitations

Because no validated measure suited our purposes, the use of an ad hoc developed questionnaire was necessary. Although a validated measure is preferable, using ad hoc, but relevant items meant that we could limit the number of items, thus, we believe, improving the response rate. Our symptom prevalences may be underestimated due to recall bias, since symptoms turning out to be harmless may probably soon be forgotten. However, we found no indication that this phenomenon was pertinent to socioeconomic status and therefore it is unlikely to have influenced our socioeconomic analyses.

The results in this paper reflect self-report and as we did not perform any clinical examinations we cannot determine the appropriateness of reporting symptoms.

Selection bias was reduced by randomly selecting participants by means of the Danish Civil Registration System. The large sample ensured a high statistical precision of our estimates with narrow confidence intervals, supported by the high participation rate.

Late responders had essentially the same prevalence of symptom reporting as immediate responders. Therefore we believe that non-responders can reasonably be expected to have a similar prevalence as well [3].

### Generalisability

Our sample is fairly representative of the Danish population according to the distribution of sex and socioeconomic factors. We calculated weighted prevalence estimates according to the Danish population. Further, as associations between health and socioeconomic status seem to be rather universal [9], it is reasonable to assume that that our results are generalisable to other Western countries.

### Comparison with existing literature

A Scottish community-based study from 1978 analysed symptom reporting and socioeconomic factors [27]. Our results cannot be compared directly, as the studies included different symptoms and had different time intervals for symptom reporting. We found different prevalence estimates for symptom reporting, which could be explained by the different time frames for symptom reporting and by the fact that children were not included in our study. For instance, we found a lower prevalence estimate for the total group with regard to coughing (6.5 vs. 15%). One reason could be that the Scottish study asked for coughing within a 2-week period only, thus including more people suffering from a simple cold.

Other studies also found that female sex were associated with more symptoms reporting [28-30]. One possible explanation could be that women have a higher bodily awareness, they pay more attention to bodily sensations, and as a consequence report symptoms more often than men [31]. Another explanation could be that women may have higher morbidity and therefore may be more familiar with recognising symptoms.

Subjects with older age had statistically significantly lower odds of reporting one or more cancer alarm symptom. The same result was found in other studies [30,32] which could be due to the interpretation of symptoms by elderly people. Elderly people, who are more likely to experience symptoms qua increasing morbidity, may not consider the symptoms to be serious, they normalise it, and therefore not necessary to report. For instance Hickey (1988) reported that elderly people have more symptoms than younger people, but when they consult doctors they tend to report fewer symptoms [33].

In line with McAteer et al. we found that those out of the workforce had significantly higher odds of reporting one or more symptoms [30]. This result may reflect a higher morbidity among this group of people [9].

A Scottish study has shown that living alone was associated with increased time before lung cancer patients consulted their doctor about symptoms [34]. Our hypothesis was that people living with a partner would report symptoms more often than singles [30], simply because they can discuss the symptom with their partner, and thereby remember the symptom. We found that subjects living alone had higher odds of reporting cancer alarm symptoms than subjects living with a partner. This pinpoints the issue that symptom registration may be a mixture of actual symptom experience and symptom interpretation.

Studies have shown that having a close experience with a cancer diagnosis is associated with greater awareness of cancer symptoms [25,26]. Likewise, we found that subjects with a cancer diagnosis had statistically significantly higher odds of reporting symptoms, which could be explained by a higher level of morbidity and by greater awareness of cancer symptoms in this group of people.

Women and those with a cancer diagnosis had statistically significantly higher odds of reporting having felt a lump in the breast. To a large extent this may be due to the fact that lumps in the breast being predominantly a gender-specific condition and because people with a cancer diagnosis pay more attention to bodily sensations. Furthermore the cancer diagnosis reported could be breast cancer, thereby giving the higher odds. Age above 60 years was statistically significantly associated with lower odds of reporting a lump in the breast. This findings are consistent with others studies indication that older people notice or report fewer symptoms [30,33]. Another explanation is that benign conditions in the breast such as fibro adenomas are found more often among younger women.

We found that subjects with high educational and income level had statistically significantly lower odds of reporting coughing for more than 6 weeks in adjusted analyses. Furthermore, we found that those out of the workforce had statistically significantly higher odds of reporting coughing. This might be explained by differences in causal factors such as tobacco smoking [35]. Future studies on symptom reporting in a population should include data on lifestyle parameters like tobacco use, alcohol consumption and diet.

Those living with a partner had statistically lower odds of reporting having seen blood in the urine. This could be due to the fact that people had discussed the symptom with their partner, interpreted it to be harmless, and then have forgotten about it.

Those out of the workforce and those with a cancer diagnosis had statistically higher odds of reporting having seen blood in the stool. It is well known that in general persons out of the workforce have a higher level of morbidity [36] and consequently this phenomenon may also contribute to more symptoms. Further, having a cancer diagnosis will make you more concerned about symptoms and bodily sensations. Women and subjects aged 40+ had statistically lower odds of reporting having seen blood in the stool. We have no qualified explanation as to why women report blood on the stools less often but we assume that the lower odds for older people are seen because they accept having different symptoms frequently – and therefore report symptoms less often.

### Implications of the study

The finding that socioeconomic and demographic determinants are associated with reporting of cancer alarm symptoms in this population-based study may help healthcare systems target preventive campaigns. However, in order to tailor campaigns these should be preceded by studies on associations between cancer alarm symptoms and healthcare consulting behaviour. Future studies should also address the impact of other factors on symptom reporting such as comorbidity, previous diseases, cancer in the respondent’s network etc.

## Conclusions

Socioeconomic and demographic determinants are associated with reporting of common cancer alarm symptoms.

### Ethical approval

According to the Scientific Ethics Committee for the County of Funen, the Biomedical Research Ethics Committee System Act does not apply to this project. The study was approved by the Danish Data Protection Agency.

## Competing interests

The authors declare that they have no competing interests.

## Authors’ contributions

BLH and HS had the original idea for the overall study. The plan of analysis for this sub-study was developed by RPS in collaboration with MSP, PVL, JS, and DEJ, who all participated in the extraction of data and interpretation of results. RPS drafted the manuscript and conducted all statistical analyses under the supervision of MSP, PVL, JS, and DEJ. All authors contributed to the final version. RPS is the guarantor.

## Data

All authors, external and internal, had full access to all of the data (including statistical reports and tables) in the study and can take responsibility for the integrity of the data and the accuracy of the data analysis.

## Data sharing

No additional data available.

## Funding

This study was funded by the Novo Nordisk Foundation. All authors are independent of funders.

## Pre-publication history

The pre-publication history for this paper can be accessed here:

http://www.biomedcentral.com/1471-2458/12/686/prepub
